# Preliminary Phytochemical, Fatty Acid, and In Vitro Antioxidant Characterization of the ‘Butko’ Olive (*Olea europaea* L.) Genotype from the Eastern Black Sea Region of Türkiye

**DOI:** 10.3390/molecules31193426

**Published:** 2026-09-26

**Authors:** Ali Murat Kesemen, Fatma Yaylacı Karahalil, Anıl Akay, Sevgi Kolaylı, Anna Kurek-Górecka

**Affiliations:** 1 Department of Hotel, Restaurant and Food Services, Artvin Vocational School, Artvin Çoruh University, 08000 Artvin, Türkiye; alimuratkesemen@artvin.edu.tr; 2 Department of Chemistry and Chemical Process Technologies, Maçka Vocational School, Karadeniz Technical University, 61080 Trabzon, Türkiye; fyaylaci@ktu.edu.tr; 3 Travel, Tourism and Entertainment Services Department, Artvin Vocational School, Artvin Çoruh University, 08000 Artvin, Türkiye; anil.akay@artvin.edu.tr; 4 Department of Chemistry, Faculty of Science, Karadeniz Technical University, 61080 Trabzon, Türkiye; 5 Department of Biochemistry, Faculty of Medicine, Nakhchivan State University, Nakhchivan 7012, Azerbaijan; 6 Department of Microbiology and Immunology, Faculty of Medical Sciences in Zabrze, Medical University of Silesia in Katowice, Jordana 19, 41-808 Zabrze, Poland; akurekgorecka@sum.edu.pl

**Keywords:** ‘Butko’ genotype, olive fruit, phenolic compounds, DPPH, FRAP, HPLC-PDA, GC-FID

## Abstract

Compositional data on the ‘Butko’ olive genotype (*Olea europaea* L.) from Artvin, Eastern Black Sea Region of Türkiye, remain limited, with no prior study integrating pomology, phenolic profiling, antioxidant capacity, and fatty acid composition. This study characterized these traits in fresh fruit. Mean fruit weight was 2.61 ± 0.20 g and length was 20.41 ± 1.20 mm. Methanolic extracts contained 561.33 ± 9.45 mg GAE/100 g total phenolic content and 51.36 ± 3.40 mg QE/100 g total flavonoid content. Ferric reducing antioxidant power was 46.20 ± 2.31 µmol FeSO_4_·7H_2_O/g, and DPPH SC_50_ was 1.15 ± 0.05 mg/mL (~0.10 mM). GC-FID analysis showed oleic acid dominated the fatty acid profile (69.37%), with linoleic (12.80%) and linolenic (0.44%) acids as minor constituents; saturated fatty acids totaled 14.82%, and squalene tentatively accounted for 1.02%. Among 26 phenolic standards screened by HPLC-PDA (excluding oleuropein), only catechin, epicatechin, luteolin, and vanillic acid were quantified. These results provide a comprehensive compositional baseline for the ‘Butko’ genotype, revealing a favorable fatty acid and phenolic makeup that highlights its potential as a functional food and valuable regional genetic resource.

## 1. Introduction

The olive (*Olea europaea* L.), a prominent member of the Oleaceae family, is a key species of the Mediterranean Basin, with substantial ecological, economic, and cultural importance [1]. With a domestication history spanning millennia, the olive tree exhibits exceptional physiological adaptation to prevailing environmental stress factors, including drought, elevated temperatures, and intense UV radiation [2]. As the cornerstone of the health-promoting Mediterranean diet, olive fruits and their derived products are globally recognized for their unique nutritional and functional properties. Olive fruit and olive oil are rich in monounsaturated fatty acids (MUFAs) and serve as a natural source of bioactive phenolic compounds, such as oleuropein, hydroxytyrosol, rutin, and luteolin. These secondary metabolites demonstrate potent antioxidant, antimicrobial, and anti-inflammatory activities [1,3,4]. Consequently, the rich phytochemical profile and cytoprotective potential of the olive have positioned it as a major focal point in bioactive natural product research, with extensive applications across pharmacology, functional foods, and the cosmetics industry [5].

As one of the primary historical centers of olive domestication, Türkiye harbors extensive genetic diversity and agricultural heritage. Favorable ecological conditions across the country have allowed both wild (*O. europaea* var. *sylvestris*) and cultivated forms to thrive over a broad geographical distribution [6,7]. Türkiye has historically ranked among the leading global producers, with commercial cultivation concentrated predominantly in the Aegean, Mediterranean, and Marmara regions, with smaller contributions from Southeastern Anatolia and the Black Sea region [8]. This ecological diversity has facilitated the emergence of numerous cultivars uniquely adapted to distinct regional microclimates [2]. The genetic diversity among these varieties directly modulates not only their pomological and morphological traits but also their secondary metabolite accumulation, antioxidant capacity, and fatty acid composition. National cultivars such as Ayvalık, Domat, and Gemlik, alongside regional genotypes with high antioxidant capacities like Halhalı from the Hatay region, exemplify this genetic richness. This extensive gene pool provides a critical foundation for investigating the plant’s phytochemical dynamics and biochemical standardization parameters [1,8].

Phytochemical and compositional traits vary widely across Turkish and Mediterranean olive genotypes. Reported total phenolic contents in olive fruit range from below 150 to over 800 mg/100 g fresh weight depending on the cultivar, ripening stage, and extraction protocol [3,5,6]. Similarly, oleic acid—the predominant fatty acid in olive lipids—typically spans from approximately 60% to 75–77% among Turkish local varieties, including Butko-group genotypes from the Eastern Black Sea region [4,9,10]. Furthermore, in vitro antioxidant capacity measured by DPPH SC50 or FRAP assays differs by nearly an order of magnitude across different genotypes and analytical methods [1,5]. Understanding this extensive variability highlights the necessity to comprehensively characterize unique regional germplasms, providing the framework to elucidate the distinct compositional profile of the ‘Butko’ genotype in the present study.

Among these valuable genetic resources, the Butko olive emerges as a distinct genotype cultivated in the historical Georgian geography, grown under the distinctive microclimate of the Yusufeli district in the Artvin province [11]. Olive cultivation in the Çoruh Valley of Artvin has been documented as a longstanding, geographically marginal activity at the northeastern limit of the species’ range in Türkiye [11], where local growers have maintained the Butko type alongside the related regional forms Otur and Sati for at least several decades [12]. Morphologically, this ecotype is distinguished from standard commercial varieties by its characteristic oval fruit shape and finely striated pit structure. From a biochemical perspective, the Butko olive demonstrates a notable oil content, with lipid accumulation averaging 25% on a fresh-weight basis. Furthermore, the organoleptic properties of its derived olive oil suggest a complex volatile profile and a distinct phenolic composition and antioxidant activity [9]. Although the pomological characteristics and fatty acid composition of the Butko, Otur, and Sati olive types from this district were documented several decades ago [10,11,12], and a subsequent survey of naturally debittered Turkish table olives reported the total phenolic content, antioxidant activity, and fatty-acid profile of Butko fruit alongside other varieties [9], these earlier studies did not report a detailed HPLC-based phenolic-compound profile or ferric-reducing antioxidant capacity, nor did they generate a pomological, phenolic, antioxidant, and fatty-acid dataset under a single, controlled recent sampling protocol. Despite its high phytochemical potential and significance for regional biodiversity conservation [13], comprehensive data integrating the specific polyphenol fractions, in vitro antioxidant capacity, and detailed fatty acid composition of the Butko genotype within a single study remain scarce.

Addressing this regional data deficiency for niche olive genotypes is essential for advancing the food-science literature on underexplored germplasm resources. Therefore, the present study combines pomological measurements, phenolic and flavonoid quantification, in vitro antioxidant assays, and fatty acid profiling into a single, integrated characterization of the Butko olive (*O. europaea*), cultivated in the specific microclimate of Artvin. Rather than treating these as isolated measurements, this multi-analytical approach was designed to link the genotype’s physical fruit characteristics to its phytochemical and lipid composition, yielding a coherent compositional profile. These findings establish a compositional baseline for this genetic resource that can inform future studies on its quality parameters, standardization, and potential value. While earlier studies [9,10,11,12,13] provided initial insights, the present work aims to deliver a broader compositional baseline for the ‘Butko’ olive from Artvin, Türkiye. Through a descriptive approach, we characterize its pomological dimensions, phenolic constituents, antioxidant capacity, and fatty acid profile under a single analytical design.

## 2. Results

Table 1 presents the pomological characteristics of the ‘Butko’ genotype. The mean fruit weight of the ‘Butko’ genotype was recorded as 2.61 ± 0.20 g per fruit. Regarding the morphological dimensions, the genotype exhibited a mean fruit length of 20.41 ± 1.20 mm and a diameter of 14.98 ± 1.05 mm.

The mesocarp and endocarp weights were found to be 1.97 ± 0.20 g and 0.64 ± 0.10 g, respectively, corresponding to a mass distribution of 75.48% flesh and 24.52% pit. The calculated pulp-per-stone ratio for this genotype is approximately 3.08.

The average oil yield recovered from fresh ‘Butko’ olive paste by n-hexane extraction was 11.50% ± 0.50 (FW). Table 2 presents the TPC and TFC of the ‘Butko’ olive genotype. The mean total phenolic content and total flavonoid content of methanolic extracts of Butko olives were determined to be 561.33 ± 9.45 mg GAE/100 g and 51.36 ± 3.40 mg QE/100 g, respectively.

The antioxidant properties of fresh ‘Butko’ olive fruits were characterized by DPPH and FRAP assays. The methanolic extract demonstrated notable radical scavenging potential with a DPPH SC_50_ of 1.15 ± 0.05 mg/mL. Consistently, the reducing power evaluated by the FRAP assay yielded 46.20 ± 2.31 µmol FeSO_4_·7H_2_O/g, reflecting the overall electron-donating capacity of the fruit matrix.

The HPLC-PDA chromatogram of the 26 authentic phenolic standards is presented in Figure S1, and the representative chromatographic profile of the methanolic ‘Butko’ olive fruit extract is shown in Figure 1. Based on the reference library used, five phenolic compounds, catechin, epicatechin, luteolin, gallic acid and vanillic acid were positively identified and quantified (Table 3). Notably, the chromatogram was dominated by a major peak that could not be definitively confirmed due to the lack of an authentic reference standard in the screened panel. However, considering the characteristic chromatographic retention behavior, UV spectral properties, and well-established olive fruit literature, this unresolved peak is tentatively presumed to be oleuropein.

The fatty acid profile of the total lipids extracted from ‘Butko’ olive fruit using hexane was determined by GC-FID following conversion to fatty acid methyl esters (FAMEs). Individual fatty acids were identified by comparing their retention times with those of authentic reference standards. As summarized in Table 4, oleic acid (C18:1) was identified as the predominant fatty acid, representing the major fraction of the total lipid content.

In this single GC-FID determination, the lipid profile of the Butko olive fruit extract was predominantly characterized by monounsaturated fatty acids (MUFAs), which constituted 70.86% of the total fatty acid composition. This fraction was overwhelmingly driven by oleic acid (18:1), the most abundant fatty acid overall, accounting for 69.37%. Minor MUFA contributors included palmitoleic (0.98%), eicosenoic (0.27%), heptadecenoic (0.16%), and (7*E*)-7-hexadecenoate (0.08%). Saturated fatty acids (SFAs) represented the second largest lipid class, comprising 14.82% of the matrix. Palmitic acid (16:0) was the dominant SFA and the third most abundant fatty acid overall, at 11.49%. Stearic acid (18:0) ranked as the second most prevalent SFA, at 2.80%. Other SFAs, such as arachidic (0.40%), isoheptadecanoic (0.07%), behenic (0.05%), and lignoceric (0.01%) acids, were detected only in negligible quantities. Polyunsaturated fatty acids (PUFAs) made up 13.27% of the total lipids, primarily consisting of linoleic acid (18:2). As the principal PUFA, linoleic acid ranked second in overall quantity, at 12.80%. Additional PUFAs, including linolenic (0.44%) and 10,13-octadecadienoic (0.03%) acids, were detected in trace amounts (<1%).

A chromatographic peak tentatively assigned to squalene accounted for 1.02% of the total detected area. Because the assignment was based on retention-time matching in a single GC-FID determination, confirmation using an authentic standard and/or GC-MS is required.

## 3. Discussion

Botanically, the olive fruit is characterized as a drupe composed of an oil-bearing fleshy mesocarp and a lignified endocarp (stone) encompassing a seed [14]. When the pomological and morphological characteristics of the ‘Butko’ genotype investigated in this study are compared with general olive data in the literature, descriptive information regarding its pomological characteristics is obtained. According to the literature, olive fruit weight varies considerably among cultivars, with a survey of 112 olive cultivars reporting a range of 1 to 10.4 g and a mean of approximately 4 g [15]. Therefore, while the ‘Butko’ genotype is well within the biological weight limits of the *O. europaea* species, it is situated close to the lower limit of the typical 3–10 g average, indicating a medium-to-small fruit profile. Standard olive fruits generally possess dimensions (both width and length) ranging from 2 to 3 cm [16]. The fruit length of the ‘Butko’ genotype aligns with the lower boundary of this standard range, whereas its diameter is slightly below it, suggesting an elongated and relatively slender fruit shape.

One of the most critical quality indicators for consumer acceptance and industrial processing is the flesh-to-pit (pulp-per-stone) ratio. Previous studies indicate that the edible pulp-per-stone ratio in olives generally ranges between 3.0 and 6.5 [16]. The ratio of 3.08 obtained from the ‘Butko’ genotype successfully falls within this critical literature range, confirming that its flesh-to-pit ratio falls within the reported range for cultivated olives. This pomological distribution suggests that, despite being classified within a relatively small to medium-sized fruit profile, the ‘Butko’ genotype may hold potential relevance for both oil extraction and table olive applications, warranting further evaluation.

When compared specifically with other local genotypes from the same Eastern Black Sea district, the ‘Butko’ fruit characterized here (mean weight 2.61 g, length 20.41 mm) is markedly smaller than the Otur genotype reported from the same Yusufeli district (fruit weight 3.93 g, length 24.77 mm) [12], consistent with Butko’s traditional characterization as the smallest-fruited member of the local Butko–Otur–Sati group, historically associated with the highest fruit count per kilogram (≈546 fruit/kg for Butko, versus ≈254 fruit/kg for Otur) [12].

The average total lipid yield obtained from fresh ‘Butko’ olives (11.50% ± 0.50%, fresh weight basis) is consistent with the oil accumulation patterns documented for early green harvest stages by Yorulmaz et al. [17]. In their study, oil yields in major Turkish cultivars (Memecik and Edremit) during the initial green maturation phase were reported within a similar range (~12–16%, fresh weight basis), subsequently increasing toward full black maturation [17]. The relatively lower yield observed in ‘Butko’ reflects typical green harvest characteristics, in which high moisture content and incomplete lipogenesis limit lipid yield compared to fully ripened fruits, alongside genotype-specific variations.

In a study conducted on olives from the Mahdia region of Tunisia, Chemlali and Neb Jmel genotypes exhibited considerable variation in total phenolic content in methanolic extracts, ranging from 139.26 to 497.48 mg/100 g and from 43.27 to 802.62 mg/100 g, respectively. These variations were strongly associated with the harvest period, with the highest TPC levels reported in October [6]. In the same study, total flavonoid content was reported to range between 160.10 and 263.04 mg catechin equivalents (CEQ) per 100 g for the Chemlali cultivar and between 95.84 and 454.70 mg CEQ/100 g for the Neb Jmel cultivar. However, in contrast to the trend observed for TPC, TFC decreased with fruit maturation, indicating an inverse relationship between flavonoid accumulation and ripening stage.

These findings highlight the considerable influence of both genotype and harvest time on phenolic and flavonoid composition and suggest that different classes of phenolic compounds may respond differently to the ripening process. In a study conducted on the Hatay (Arsuz) region of Türkiye, the Halhalı olive cultivar exhibited a mean total phenolic content of 270.99 ± 0.30 µg GAE/mg extract in methanolic extracts [1]. However, the results were expressed on an extract basis and thus do not provide direct information on the phenolic content per unit weight of fresh fruit, limiting direct comparison with studies reporting values on a fruit basis. In a study conducted on the Memecik and Edremit olive cultivars, which are widely produced in Türkiye, the total phenolic content was reported to range between 374 and 810 mg GAE/kg [17]. In another study, the total phenolic content of olive fruit was reported as 245.2 ± 0.65 mg oleuropein equivalents (OLE) per g [5]. However, direct comparison of these values with those obtained for Butko olives remains challenging due to differences in analytical conditions, including the standards used (e.g., oleuropein equivalents vs. gallic acid equivalents), extraction solvents, and methodological approaches. For a more accurate and meaningful comparison of total phenolic and flavonoid contents, it is essential that studies employ comparable analytical protocols and report results using consistent units and reference standards. Therefore, a direct quantitative comparison with the present findings is not fully feasible. Nevertheless, considering the overall trends reported in the literature, the measured TPC indicates a substantial phenolic content under the extraction and assay conditions used in the present study. Results have also been expressed as tannic acid equivalents [3], gallic acid equivalents [1], or oleuropein equivalents [5] in other studies, which complicates direct comparison. In a study conducted on the same Butko olive cultivar, the total phenolic content was reported as 152.09 ± 7.46 mg GAE/100 g [9]. The substantial TPC observed here (561.33 ± 9.45 mg GAE/100 g) can be attributed to a combination of agro-climatic and analytical parameters. Local climatic dynamics, harvest timing, and physiological ripening status strongly govern secondary metabolite synthesis in olive fruit. Additionally, methodological discrepancies among studies, particularly regarding the choice of extraction solvents, sample-to-solvent ratios, and extraction protocols, likely account for these quantitative variations. It is well established that total phenolic content is strongly influenced by fruit maturity, typically exhibiting a declining trend as ripening progresses [2,5]. Therefore, differences in ripening stage are likely a major contributor to the variability observed between the present findings and those reported in the literature. In addition, inconsistencies among published results may also arise from differences in analytical methodologies and calibration approaches, further complicating direct comparisons across studies.

The DPPH test measures the ability of extracts to donate hydrogen or electrons to DPPH, a stable free radical, while the FRAP test reflects the capacity of antioxidant compounds to reduce the Fe(III)-TPTZ complex to the Fe(II)-TPTZ form under low pH conditions. Consistent with our findings, DPPH radical scavenging activity values have been reported as 9.47 mg/mL [1] and 0.73 ± 0.19 mg/mL [5], indicating variability across studies likely related to differences in experimental conditions and extract composition. In a related study, the SC_50_ values for DPPH radical scavenging activity were reported as 0.058 ± 0.01 mg/mL for ascorbic acid (vitamin C) and 0.75 ± 0.06 mg/mL for BHT [5], highlighting the strong antioxidant capacity of these standard reference compounds in comparison to plant-derived extracts. DPPH radical scavenging activity is more commonly expressed in the literature as percentage inhibition; for instance, values ranging between 85% and 91% have been reported for Memecik and Edremit olive cultivars [17]. However, direct comparison with the present results is not fully possible, as different studies employ varying expression formats, such as SC_50_ values or percentage scavenging activity, as well as differences in assay conditions and extract preparation. Therefore, while the reported data indicate measurable antioxidant activity, methodological inconsistencies limit precise quantitative comparison across studies. For broader context, various plant-derived natural products have also been evaluated for DPPH radical scavenging capacity; for example, SC_50_ values of 0.35 mg/mL for green tea, 2.52 mg/mL for blueberry, 0.29 mg/mL for aronia berry, and 24.77 mg/mL for chestnut honey have been reported in the literature [18]. In the present study, the ‘Butko’ olive extract exhibited an SC_50_ value of 1.15 ± 0.05 mg/mL, placing it within this broader range and indicating measurable radical-scavenging activity; however, such cross-matrix comparisons remain indicative rather than direct, given the substantial differences in matrix composition, extraction conditions, and assay parameters across studies. Similarly, to better interpret the FRAP value obtained from methanolic olive fruit extracts (46.20 ± 2.31 µmol FeSO_4_·7H_2_O/g), it is necessary to compare these results with those of other natural products. Turkish propolis, for example, has been reported to exhibit a FRAP value of 328.61 µmol FeSO_4_·7H_2_O/g [19].

Olive fruits possess a diverse phenolic profile that determines their nutritional quality and antioxidant potential. According to the literature, the most abundant secondary metabolite in the fruit is the secoiridoid oleuropein [2,5]. During the maturation process, hydrolytic enzymes (such as glucosidases) break down oleuropein into hydroxytyrosol, a compound with remarkably high biological activity and antioxidant capacity. This degradation process demonstrates the direct impact of the ripening stage on the phenolic composition of the fruit [6]. In addition to these primary components, olives contain notable amounts of phenolic alcohols like tyrosol, major flavonoids such as luteolin, apigenin, and rutin, as well as various phenolic acids, particularly caffeic, *p*-coumaric, gallic, and vanillic acids. The concentration and distribution of these metabolites vary significantly depending on the genotype, the developmental stage at harvest, and whether the phenolic fractions are in free or bound forms [6]. The measured total phenolic and flavonoid values indicate the presence of a substantial phenolic fraction under the assay conditions used. This phytochemical profile suggests potential relevance of the ‘Butko’ genotype for future food-quality or bioactive-compound studies.

Oleic acid, the primary monounsaturated fatty acid (MUFA) of the olive oil lipid profile, forms the dominant fraction in all studied olive oil varieties; based on the literature, its concentration typically varies between 60.00% and 72.77% [4,9,20]. The oleic acid content of the hexane-extracted fruit lipid fraction reported here (69.37%) is somewhat lower than the 74.92% previously reported for Butko olive oil from the same district [10]; this difference may reflect differences in harvest year, ripening stage, or the specific lipid matrix analyzed (fruit lipid extract in the present study versus extracted olive oil in [10]). The palmitic acid content recorded in this study falls near the lower bound of the typical range of 12.00% to 16.80% reported for diverse olive varieties [4,9]. The stearic acid content falls within previous literature establishing a normal range of 2.50% to 3.48% for olive oils [9,21,22]. The linoleic acid proportion falls within the established fluctuation range of 9.47% to 19.15%, which is known to be highly dependent on the specific olive cultivar [4,9,21].

Squalene is a minor unsaponifiable constituent of olive oil and has been associated with its compositional and oxidative characteristics [23]. The overall fatty acid profile is dominated by monounsaturated fatty acids, particularly oleic acid, followed by polyunsaturated and saturated fatty acids, a distribution broadly consistent with typical olive oil lipid profiles [24,25].

## 4. Materials and Methods

### 4.1. Samples

Olive (*Olea europaea* L.) samples, authenticated as the local ‘Butko’ genotype, were harvested in October 2025 from an orchard in Demirkent village (approx. 40.88° N, 41.73° E), located in the Yusufeli district of Artvin province, Türkiye (Figure 2). Botanical identification and cultivar verification were performed by Dr. Hayal Akyıldırım Beğen (Artvin Çoruh University, Artvin, Türkiye). The sampling was conducted at the fully ripe, black skin coloration stage according to traditional regional harvesting practices; however, an objective maturity index (e.g., the Jaén index 0–7) was not formally quantified for this lot. To achieve a representative field batch, fruits were hand-picked and pooled from multiple healthy, adult trees (approx. 5–8 trees) within the same grove to constitute a single composite sample. Upon collection, representative photographs of the harvest lot were documented, and the fresh fruits were immediately transferred to the laboratory under cold chain conditions (+4 °C). Analytical extractions and measurements commenced within 24 h of harvest. From this homogenized composite pool, three independent analytical extracts were prepared and analyzed in parallel (representing extraction/analytical replicates rather than independent biological replicates from separate trees). Given that this sampling was restricted to a single harvest event from a single micro-location, this dataset represents a preliminary baseline; systematic multi-year and multi-orchard samplings with standardized ripening indices are planned to fully characterize inter-annual and spatial variability across the wider ‘Butko’ population.

### 4.2. Pomological Characterization

Pomological characterization of the ‘Butko’ genotype was carried out on 30 freshly harvested, visually undamaged fruits (*n* = 30), randomly selected from the sample material described above. Briefly, each fruit was individually weighed on an analytical balance (precision ± 0.01 g), after which the flesh (mesocarp) was manually separated from the pit (endocarp) and the two fractions were weighed separately. Fruit length and diameter, flesh thickness, and pit length and diameter were then recorded for each fruit using a digital caliper (precision ± 0.01 mm). The flesh and pit proportions reported in Table 1 were subsequently calculated as the percentage ratio of the mean flesh weight and the mean pit weight, respectively, to the mean total fruit weight. The estimated fruit count per kilogram reported in Table 1 was derived as 1000 divided by the mean individual fruit weight, rather than from an independent count of a 1 kg bulk sample.

### 4.3. Extraction

Lipids were extracted using a Soxhlet apparatus in accordance with the AOCS Official Method [26]. Fresh green olives were pitted and homogenized into a uniform paste. An aliquot of approximately 10 g was weighed and oven-dried at 105 °C for 24 h to eliminate moisture, followed by cooling to room temperature in a desiccator. The dried sample was placed into a cellulose extraction thimble and extracted with n-hexane in a Soxhlet extractor for 6–8 h. The solvent was subsequently removed under reduced pressure using a rotary evaporator at 40 °C. The recovered oil was determined gravimetrically, and the oil yield was expressed as a mass fraction of the initial fresh weight (%, w/w, fresh basis). The obtained oil was stored under refrigeration prior to fatty acid profiling.

Phenolic compounds were recovered following the procedure described by Yorulmaz et al. [17] with minor modifications. Briefly, 1.5 g of pitted, homogenized olive paste was extracted with 20 mL of methanol/water (80:20, v/v) in an ultrasonic bath for 10 min at room temperature. The mixture was filtered through Whatman No. 40 filter paper. To eliminate residual lipophilic constituents, 20 mL of n-hexane was added to the filtrate and thoroughly shaken. After complete phase separation, the upper hexane layer was discarded. The lower hydromethanolic phase was collected, passed through a 0.45 µm PTFE syringe filter, and stored at −18 °C until chromatographic and spectrophotometric analyses.

### 4.4. Determination of Total Phenolic Content (TPC)

The total phenolic content of the olive extracts was determined using the Folin–Ciocalteu colorimetric method [27]. Briefly, 0.5 mL of the methanolic extract was mixed with 2.5 mL of 0.20 N Folin–Ciocalteu reagent. After standing for 5 min, 2 mL of 10% (*w*/*v*) sodium carbonate aqueous solution was added to the mixture. The samples were then incubated for 2 h at room temperature in the dark. Following the incubation period, 5 mL of distilled water was added to the mixture, and the absorbance was measured at 725 nm using a UV-VIS spectrophotometer (Shimadzu UV mini-1240, Kyoto, Japan). Gallic acid was used to construct the standard calibration curve, and the results were expressed as mg GAE/100 g FW.

### 4.5. Determination of Total Flavonoid Content (TFC)

The total flavonoid content of the extracts (prepared as described in Section 4.3) was evaluated following the protocol of Fukumoto and Mazza [28]. A 250 µL volume of the extract was mixed thoroughly with 1.25 mL of methanol, 50 µL of 10% Al(NO_3_)_3_, and 50 µL of 1 M CH_3_COONH_4_. After a 50 min incubation period at room temperature, the absorbance of the reaction mixture was recorded at 415 nm. Total flavonoid concentrations were determined from the linear regression equation mg QE/100 g from y = 4.8243 × −0.008, R2:0.999 and presented as mg QE/100 g.

### 4.6. Determination of DPPH Radical Scavenging Activity

The DPPH (2,2-diphenyl-1-picrylhydrazyl) radical scavenging activity was determined by a spectrophotometric assay [29]. An aliquot of the extract (1.0 mL) was mixed with 1.0 mL of a 0.04 mg/mL DPPH solution (Sigma-Aldrich, Darmstadt, Germany). Following a 45 min incubation period in the dark at a constant temperature of 25 °C, the absorbance of the mixture was measured at 517 nm. The half-maximal scavenging concentration (SC_50_) was established by testing six varying concentrations of the methanolic extract. The absorbance readings were recorded at 517 nm, and radical scavenging inhibition was calculated relative to the untreated DPPH control. The concentration of the extract providing 50% inhibition (${\mathrm{SC}}_{50}$) was determined by plotting scavenging percentages across a range of serial concentrations and interpolating the value from the resulting linear regression curve.

### 4.7. Determination of Ferric Reducing Antioxidant Power (FRAP)

The ferric reducing antioxidant power (FRAP) assay was used to determine total antioxidant capacity [30]. The FRAP reagent was prepared by mixing 10 mM TPTZ (2,4,6-tri(2-pyridyl)-*s*-triazine) in 40 mM HCl (6 mL) with 300 mM acetate buffer at pH 3.6 (60 mL) and 20 mM ferric chloride (6 mL). An aliquot of 75 µL of the extract solution and 225 µL of deionized water were added to 2.25 mL of the FRAP reagent, and the absorbance was measured at 593 nm after incubation of the mixture at 37 °C for 30 min. FeSO_4_·7H_2_O was used as a reference standard to construct the calibration curve (31.25–1000 µM). FRAP values were expressed as µmol FeSO_4_·7H_2_O equivalent per g fresh weight of sample.

### 4.8. Determination of Phenolic Composition Using HPLC-PDA

The qualitative and quantitative profiling of 26 distinct phenolic compounds in the olive fruit extracts was conducted using a reversed-phase high-performance liquid chromatography (RP-HPLC) system coupled with a photodiode array (PDA) detector (Shimadzu Corporation LC 20AT-SPD M30A, Kyoto, Japan). The analyses were performed on olive samples prepared as 80% methanolic extracts. Chromatographic separation was achieved on an analytical C18 column (250 mm × 4.6 mm, 5 µm particle size) supplied by GL Sciences, with the column oven temperature maintained at 30 °C. The analysis followed the validated methodology previously described by Kara and Birinci [31]. A gradient elution profile was applied using a two-solvent system consisting of 70% aqueous acetonitrile (mobile phase A) and 2% aqueous acetic acid (mobile phase B), following the gradient program: 0 min, 82% B; 5 min, 81% B; 10 min, 73% B; 14 min, 62% B; 25 min, 35% B; 40 min, 10% B; 40.01 min, 82% B; 50 min, 82% B (total analysis time 50 min, including column re-equilibration). The flow rate was kept constant at 1.0 mL/min, and the injection volume was set to 20 µL for all standards and extracted samples, with the autosampler cell temperature maintained at 20 °C. Standards were prepared by first dissolving them in a small volume of 100% methanol and then adjusting to final volume with 50% methanol. Chromatograms were monitored at four wavelengths (250, 280, 320, and 360 nm), selected according to the absorption maxima of the 26 reference standards used for compound identification and quantification: protocatechuic acid, *p*-hydroxybenzoic acid, vanillic acid, rutin, ellagic acid, and daidzein at 250 nm; gallic acid, catechin hydrate, epicatechin, syringic acid, *t*-cinnamic acid, naringenin, hesperetin, chrysin, and pinocembrin at 280 nm; chlorogenic acid, caffeic acid, *p*-coumaric acid, ferulic acid, apigenin, and caffeic acid phenethyl ester (CAPE) at 320 nm; and myricetin, luteolin, quercetin, rhamnetin, and galangin at 360 nm. Compounds in the olive fruit extracts were identified by comparing their retention times and PDA spectra with those of the authentic standards and were quantified from external calibration curves constructed for each standard.

The samples were subjected to liquid–liquid extraction twice at room temperature. Each sample was extracted first with diethyl ether (10 mL, then 5 mL) with continuous shaking (200 rpm, 15 min), followed by an identical double-extraction cycle using ethyl acetate. The collected organic phases were pooled and evaporated to dryness under reduced pressure at 40 °C. The resulting residue was redissolved in 2 mL of HPLC-grade methanol, passed through a 0.45 µm membrane filter, and directly injected into the RP-HPLC-PDA system [32].

### 4.9. Determination of Fatty Acid Composition Using Gas Chromatography–Flame Ionization Detection (GC-FID)

The derivatization of the samples into fatty acid methyl esters (FAMEs) was carried out using n-hexane and methanolic potassium hydroxide (KOH), strictly following the ISO 5509 standard method (ISO 5509:2000). The FAME profiles were subsequently analyzed using a gas chromatography (GC) system (Shimadzu GC-2010, Kyoto, Japan) equipped with a flame ionization detector (FID); together, these constitute the GC-FID system used throughout this study. Chromatographic separation was achieved on a Teknokroma TR-CN100 fused silica capillary column (60 m length × 0.25 mm internal diameter × 0.20 µm film thickness; P/N TR 882162), following the method [17].

The operating conditions were optimized as follows: the temperatures of both the injection port and the detector were set at 260 °C. Nitrogen (N_2_) was utilized as the carrier gas with a constant column flow rate of 1.51 mL/min and a total flow rate of 80 mL/min. The samples were injected with a split ratio of 1:40, operating under an inlet pressure of 200 kPa. The oven temperature program was initiated at 90 °C, held for 7 min, then ramped at a rate of 5 °C/min to reach a final temperature of 240 °C, where it was maintained isothermally for 15 min. Fatty acid methyl esters were identified by comparing their retention times with those of authentic FAME standards. Squalene and alpha-farnesene were not included in the FAME fraction and were only tentatively assigned from their chromatographic retention behavior; their identities require confirmation using authentic standards and/or GC-MS. The results were obtained from a single analytical determination and are therefore presented as descriptive relative chromatographic percentages without SD. Relative FAME content (%) was calculated by uncorrected area normalization, i.e., expressing the peak area of each compound as a percentage of the total peak area of all resolved chromatographic peaks in the run, comprising the 14 identified FAMEs (Table 4A) together with the two tentatively assigned non-FAME lipophilic peaks, squalene and alpha-farnesene (Table 4B); no theoretical flame-ionization-detector response (correction) factors were applied, consistent with common practice for routine FAME profiling by GC-FID.

### 4.10. Statistical Analysis

All spectrophotometric assays (TPC, TFC, DPPH, and FRAP) and HPLC-PDA phenolic determinations are reported as the mean ± standard deviation (SD) of analytical replicates (*n* = 3) prepared from the homogenized composite batch. Pomological dimensions are expressed as the mean ± SD (*n* = 30), with flesh-to-stone proportions and fruit counts calculated as derived values. The fatty acid composition determined by GC-FID corresponds to a single chromatographic analysis and is presented descriptively without SD. Descriptive statistics were employed throughout, with no comparative inferential analyses performed due to the single-genotype, descriptive design of the study.

## 5. Conclusions

This study provides an integrated compositional characterization of the local ‘Butko’ olive genotype from the Eastern Black Sea Region of Türkiye. The fruit exhibits a favorable flesh-to-pit ratio, strong in vitro antioxidant activity, and a polyphenol-rich profile dominated by catechin and luteolin alongside a putative major secoiridoid constituent. Furthermore, the lipid fraction is predominantly composed of oleic acid (~69.4%), aligning well with standard nutritional and commercial benchmarks for cultivated olives. While these baseline data highlight the potential of ‘Butko’ as a valuable regional genetic resource for table and oil applications, multi-season field sampling, objective maturity scoring, and mass spectrometric confirmation of the uncharacterized phenolic fraction are required to fully support its valorization and commercial preservation.

## Figures and Tables

**Figure 1:**
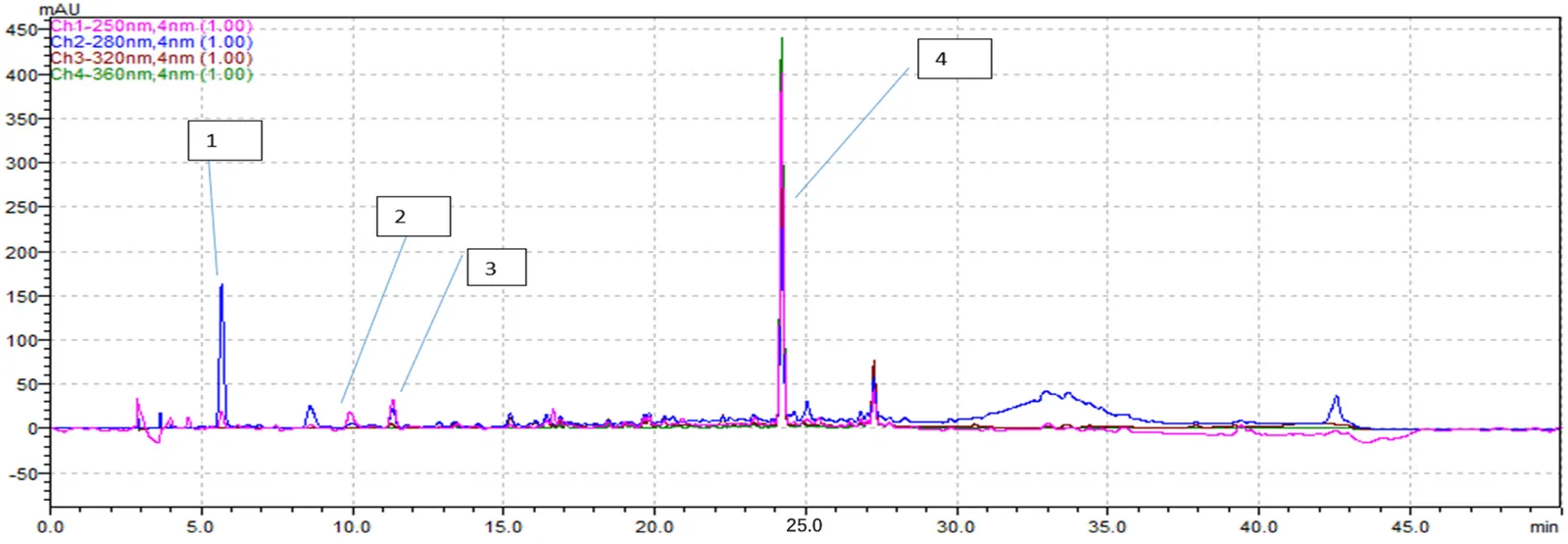
HPLC-PDA chromatographic profile of phenolic compounds in ‘Butko’ olive fruit extract. 1. Catechin hydrate 2. Gallic acid, 3. Epicatechin, 4. Vanillic acid, 5. Luteolin.

**Figure 2:**
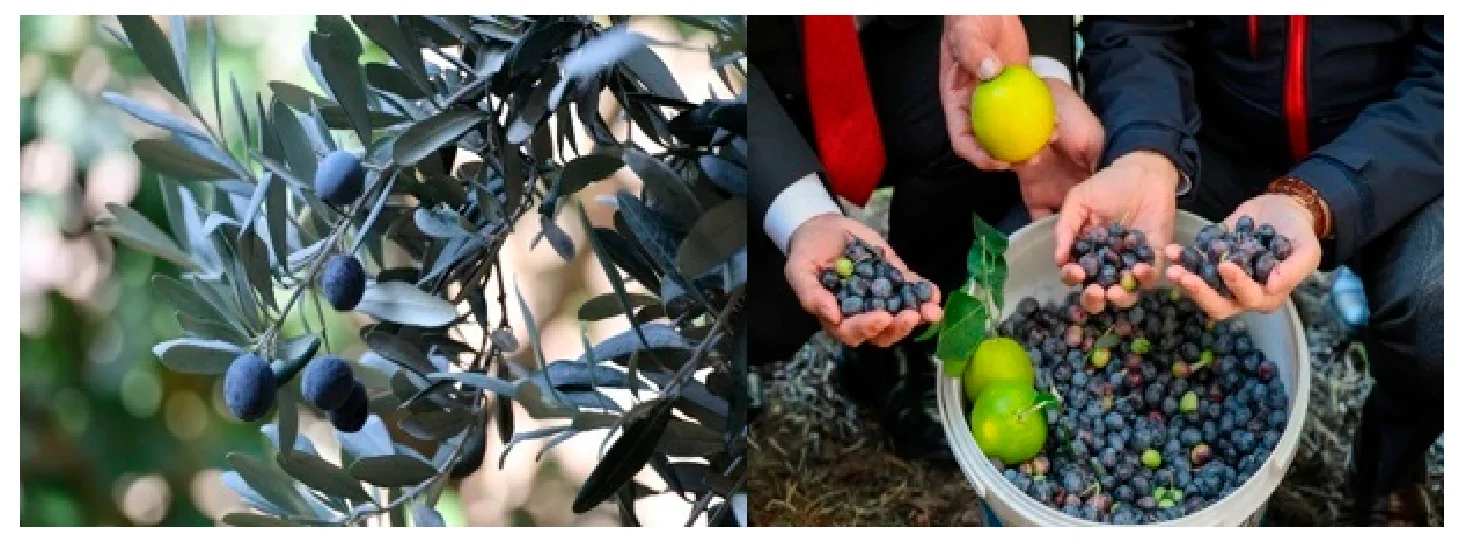
‘Butko’ genotype olive fruit (*Olea europaea* L.) from the Artvin region.

**Table 1:** Pomological characteristics and derived indices of the ‘Butko’ olive fruit.

Parameter	Value
Mean fruit weight (g)	2.61 ± 0.20
Estimated fruit count (per kg)	~383
Mean flesh weight (g)	1.97 ± 0.20
Mean pit weight (g)	0.64 ± 0.10
Flesh proportion (%)	75.48
Pit proportion (%)	24.52
Mean fruit length (mm)	20.41 ± 1.20
Mean fruit diameter (mm)	14.98 ± 1.05
Mean flesh thickness (mm)	3.70 ± 0.50
Mean pit length (mm)	14.23 ± 1.04
Mean pit diameter (mm)	7.76 ± 0.50

Values are expressed as mean ± SD (*n* = 30), except flesh and pit proportion and estimated fruit count, which are derived values (see Section 4.2). Flesh thickness was measured at the equatorial plane of each sectioned fruit, and fruit diameter was measured externally (skin intact) at the same equatorial plane; both are independently averaged, population-level measurements (*n* = 30) rather than paired single-fruit measurements, which accounts for the minor arithmetic gap between pit diameter + 2× flesh thickness and the reported mean fruit diameter (0.18 mm).

**Table 2:** Total phenolic content, total flavonoid content, and antioxidant properties of the ‘Butko’ olive fruit.

Assay (Unit)	Mean ± SD
Total Phenolic Content (mg GAE/100 g FW)	561.33 ± 9.45
Total Flavonoid Content (mg QE/100 g FW)	51.36 ± 3.40
FRAP (µmol FeSO_4_·7H_2_O/g FW)	46.20 ± 2.31
DPPH SC_50_ (mg/mL)	1.15 ± 0.05

TPC, TFC, FRAP and DPPH values are expressed as mean ± SD of three extracts prepared from a single pooled composite sample (*n* = 3). GAE: gallic acid equivalents; QE: quercetin equivalents.

**Table 3:** Phenolic compounds identified and quantified in ‘Butko’ olive fruit by HPLC-PDA.

Phenolic Compound	Content (mg/100 g FW)
Catechin	26.09 ± 0.05
Epicatechin	0.90 ± 0.02
Luteolin	8.31 ± 0.50
Vanillic acid	0.96 ± 0.04
Gallic acid	4.50 ± 0.30
Protocatechuic acid	ND
p-Hydroxybenzoic acid	ND
Rutin	ND
Ellagic acid	ND
Daidzein	ND
Syringic acid	ND
t-Cinnamic acid	ND
Naringenin	ND
Hesperetin	ND
Chrysin	ND
Pinocembrin	ND
Chlorogenic acid	ND
Caffeic acid	ND
p-Coumaric acid	ND
Ferulic acid	ND
Apigenin	ND
Caffeic acid phenethyl ester (CAPE)	ND
Myricetin	ND
Quercetin	ND
Rhamnetin	ND
Galangin	ND

Values for the five quantified compounds are mean ± SD of three extracts prepared from a single pooled composite sample (*n* = 3). ND: not detected/below the identification threshold of the 26-standard panel used.

**Table 4:** GC-FID chromatographic profile of the ‘Butko’ olive fruit lipid extract, obtained from a single analytical determination. *(**A**) Fatty acid methyl esters (FAMEs) identified using authentic FAME standards. (**B**) Tentatively assigned non-FAME lipophilic compounds*.

**(A)**			
**No.**	**RT (min)**	**Name of the Compound**	**Relative FAME Content (%)**
1	16.623	Hexadecanoic acid, methyl ester	11.49
2	17.352	Methyl (7*E*)-7-hexadecenoate	0.08
3	17.525	Methyl palmitoleate	0.98
4	18.185	Methyl isoheptadecanoate	0.07
5	19.113	*cis*-10-Heptadecenoic acid, methyl ester	0.16
6	20.144	Methyl stearate (18:0)	2.80
7	21.108	Oleic acid, methyl ester	69.37
8	21.790	10,13-Octadecadienoic acid, methyl ester	0.03
9	22.161	Linoleic acid, methyl ester	12.80
10	22.944	Eicosanoic acid, methyl ester	0.40
11	23.496	Linolenic acid, methyl ester	0.44
12	23.745	*cis*-11-Eicosenoic acid, methyl ester (20:1)	0.27
13	25.738	Docosanoic acid, methyl ester (22:0)	0.05
14	28.357	Tetracosanoic acid, methyl ester	0.01
**(B)**			
**No.**	**RT (min)**	**Tentatively Assigned Compound**	**Relative Chromatographic Area (%)**
1	8.346	alpha-Farnesene	0.005
2	27.066	Squalene (C_30_H_50_)	1.02

Results are from a single analytical determination and are presented descriptively (no SD). RT: retention time. The relative percentages in panel (A) sum to 98.95%; the remaining 1.05% is accounted for, within the rounding precision of area-percentage reporting, by the two tentatively assigned non-FAME lipophilic peaks in panel (B) (squalene, 1.02%, and alpha-farnesene, 0.005%), which were excluded from the FAME fraction because they are not fatty acid methyl esters. Summing panels (A) and (B) (98.95% + 1.02% + 0.005%) gives 99.98% of the total chromatographic peak area, consistent with 100% within instrumental and rounding tolerance.

## Data Availability

The original contributions presented in this study are included in the article. Further inquiries can be directed to the corresponding author.

## Supplementary Materials

The following supporting information can be downloaded at: https://www.mdpi.com/article/10.3390/molecules31193426/s1, Figure S1: HPLC-PDA chromatogram of 26 phenolic standards.1. Gallic acid, 2. Protocatechuic acid, 3. Chlorogenic acid, 4. Catechin hydrate, 5. p-OH Benzoic acid, 6. Epicatechin, 7. Caffeic acid, 8. Vanillic acid, 9. Syringic acid, 10. p-Coumaric acid, 11. Rutin, 12. Ellagic acid, 13. Ferulic acid, 14. Myricetin, 15. Daidzein, 16. Luteolin, 17. Quercetin, 18. t-Cinnamic acid, 19. Naringenin, 20. Apigenin, 21. Hesperetin, 22. Rhamnetin, 23. Chrysin, 24. Pinocembrin, 25. CAPE, 26. Galangin.
