# Supplementary material for: Preliminary Phytochemical, Fatty Acid, and In Vitro Antioxidant Characterization of the ‘Butko’ Olive (Olea europaea L.) Genotype from the Eastern Black Sea Region of Türkiye
Source: Molecules. 2026 Sep 26;31(19):3426. doi: 10.3390/molecules31193426 (PMC13666110; doi:10.3390/molecules31193426)
Supplement: Supplementary file 1 [file molecules-31-03426-s001.zip › molecules-4543288-supplementary.pdf]

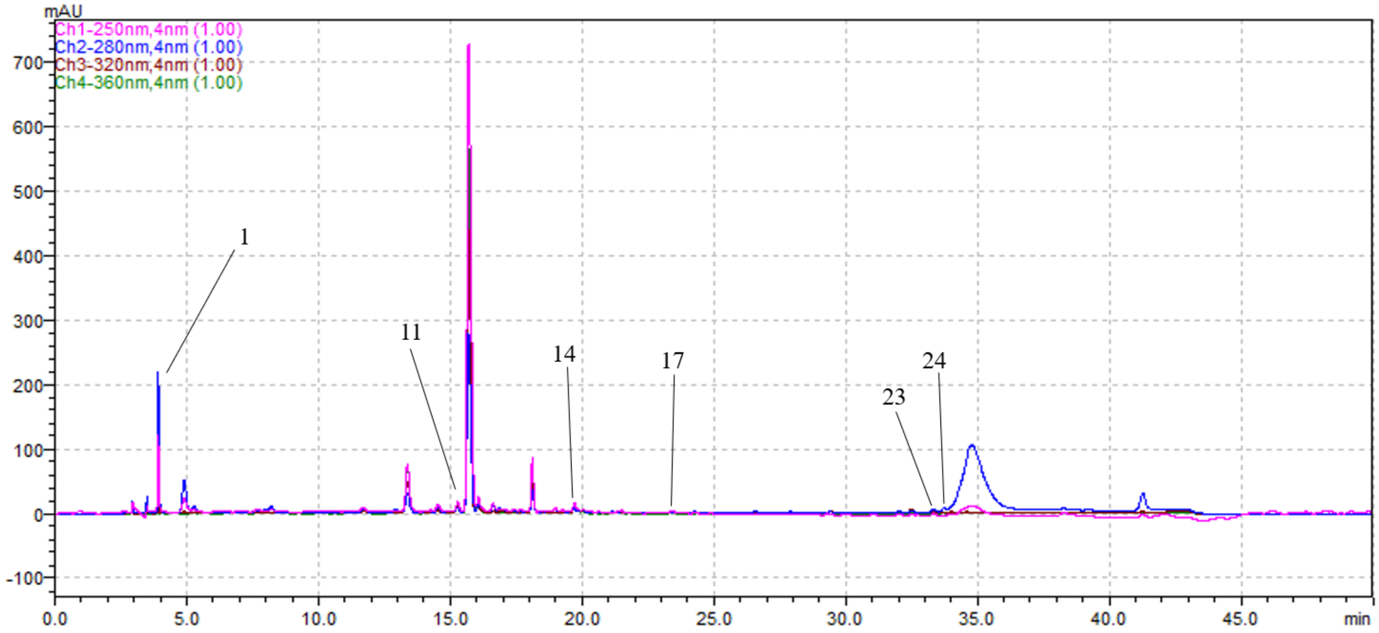

Figure S1. HPLC-PDA chromatogram of 26 phenolic standards.1. Gallic acid, 2. Protocatechuic acid, 3. Chlorogenic acid, 4. Catechin hydrate, 5. *p*-OH Benzoic acid, 6. Epicatechin, 7. Caffeic acid, 8. Vanillic acid, 9. Syringic acid, 10. *p*-Coumaric acid, 11. Rutin, 12. Ellagic acid, 13. Ferulic acid, 14. Myricetin, 15. Daidzein, 16. Luteolin, 17. Quercetin, 18. *t*-Cinnamic acid, 19. Naringenin, 20. Apigenin, 21. Hesperetin, 22. Rhamnetin, 23. Chrysin, 24. Pinocembrin, 25. CAPE, 26. Galangin
